# Influence of metformin intake on serum vitamin B12 levels in patients with type 2 diabetes mellitus

**DOI:** 10.1371/journal.pone.0279740

**Published:** 2022-12-30

**Authors:** Saad Al-Fawaeir, Ibrahim Al-Odat

**Affiliations:** Department of Medical Laboratory Science, Jadara University, Irbid, Jordan; Texas A&M University College Station, UNITED STATES

## Abstract

**Objective:**

To describe the prevalence of vitamin B12 deficiency among Jordanian patients with type 2 diabetes mellitus treated with metformin and to compare the findings with those who did not receive metformin.

**Design and method:**

Total 155 patients with type 2 diabetes mellitus, aged between 48 and 82 years were enrolled in the current study. They were divided into two groups; the first (n = 120) was treated with metformin while the second (n = 35) was not. Patients’ demographics (age, gender, duration of type 2 diabetes mellitus, smoking status), medication parameters (daily dosage and duration of metformin therapy), and biochemical parameters (hemoglobin level, mean corpuscular volume (MCV), serum vitamin B12, and folate level) were recorded. Definite deficiency was defined as serum vitamin B12 levels of < 150 pg/ml, whereas < 200 pg/ml indicated possible deficiency.

**Results:**

The mean serum ± standard deviation (SD) vitamin B12 level was significantly lower in patients who were treated with metformin (268.5 ± 35.8 vs. 389.5 ± 29.8 pg/ml, p = 0.029). The metformin group had significantly higher prevalence of definite deficiency (32% vs. 9%, p < 0.02) and possible deficiency (48% vs. 30%, p < 0.02). Within the metformin group, the mean serum ± SD vitamin B12 level was significantly lower in those on high dosage (175.2 ± 30.5 vs. 315.6 ± 37.8 pg/ml, p < 0.001). MCV (μm^3^) levels ± SD were higher in the metformin group (87.5 ± 2.9 vs. 83.7 ± 2.4) with no statistical significance.

**Conclusion:**

There is a significant association between metformin intake and vitamin B12 deficiency. Serum vitamin B12 levels should be checked by physicians and serial monitoring is necessary in patients who are treated with metformin.

## Introduction

The most common form of diabetes in the world is type 2 diabetes mellitus, affecting 85–90% of all people with diabetes. While old people, adults, and youngsters are usually affected by type 2 diabetes mellitus, children are also known to get it. It results from a combination of genetic and environmental factors. There is a strong genetic predisposition, and the risk is greatly increased when combined with lifestyle conditions such as high blood pressure, overweight or obesity, insufficient physical activity, and poor diet. Type 2 diabetes can often initially be treated by healthy diet and regular physical activity [1, 2]. However, over time most patients with type 2 diabetes also need oral medication and many require insulin treatment.

Metformin is an oral anti-diabetic drug of the biguanide class; it is the first-line drug of choice for the treatment of type 2 diabetes [3]. Metformin is one of the anti-hyperglycemic agents associated with improvement in cardiovascular morbidity [4] that causes death in type 2 diabetes mellitus patients [5].

Metformin intake has a few disadvantages. It prompts vitamin B12 malabsorption; which increases the risk of development of vitamin B12 deficiency [6–8]. In addition, treatment with metformin may cause decrease in folic acid concentration [9]. Decrease in both vitamin B12 and folate concentration leads to increase in homocysteine concentration, an independent risk factor for cardiovascular disease [10]. Reduced cognitive function and Alzheimer’s disease were also linked to low level of serum vitamin B12 in older patients [11, 12] Association between vitamin B12 deficiency and microangiopathic hemolysis [13] and narcolepsy [14] were also reported.

Several studies confirming the association between metformin intake and vitamin B12 deficiency in type 2 diabetes have subsequently been published. However, few of these studies assessed the frequency of vitamin B12 deficiency in type 2 diabetes treated with metformin.

The main aim of this study is to assess the prevalence of vitamin B12 deficiency in type 2 diabetes patients in a Jordan population sample and to compare the prevalence of vitamin B12 deficiency between metformin treated and non-treated patients.

## Methods and material

### Ethical approval

Research ethical approval has been obtained for this study from the Jordanian Ministry of Health.

### Patients

The subjects of this study were identified from the outpatient internal medicine clinic in specialty clinics of Tohama Medical Center, Zarqa city in Jordan during the period between April 2020 and March 2021. In order to participate in the current study, a written consent was obtained, in a five minute face-to-face interview, from each patient after full explanation of the purpose and nature of all procedures used. In total, 120 patients aged 48 to 82 (mean 65; 28 females, 92 males) with type 2 diabetes mellitus and a control group of 35 patients (24 male, 11 female) with type 2 diabetes mellitus not on metformin therapy or placebo were recruited. Patients’ demographics (sex, age, duration of type 2 diabetes mellitus, smoking status, and daily dosage and duration of metformin) were recorded. Blood samples were collected for serum vitamin B12, folate, hemoglobin A1c, hemoglobin, and MCV. All these tests were done in Teriaq Alrooh medical laboratory in Zarqa city. Two reference levels for vitamin B12 were used: border deficiency (< 150 pg/ml) and possible deficiency (< 200 pg/ml). The subjects in both groups were enrolled in the study voluntarily.

### Criteria

The following cases were excluded in the present study: chronic renal insufficiency, history of pernicious anemia, gastrectomy, prior bariatric surgery, daily oral or injection vitamin B12 supplementation, prior ileum resection, and Crohn’s disease.

### Anthropometric measurements

For all study subjects, weight was recorded to the nearest 0.5 g, and height was measured to the nearest 0.5 cm. The body mass index was calculated for every individual as weight in kilogram divided by the height in meters squared and categorized as normal (18–25) or overweight (25–30) or obese (> 30) as previously reported [9].

### Biochemical measurements

Serum vitamin B12 and serum folate were determined using a Cobas e 411 analyzer (Roche Diagnosis GmbH, Mannheim, Germany; normal range 190–945 pg/ml and 4.6–12.7 pg/ml for vitamin B12 and folate, respectively).

Hemoglobin and MCV were determined using Sysmex k1000 hematology analyzer (Tao electronics, Japan).

### Statistical analysis

All statistical analyses were performed using Statistical Package for the Social Sciences (SPSS) for windows 15.0 (SPSS Inc. Headquarters, Chicago, III., USA) software program and Microsoft Excel 2007 program. P < 0.05 was considered to be statistically significant.

## Results

The mean patient age of metformin and non-metformin groups were 63.8 ± 6.9 and 65.9 ± 7.3 years, respectively. The two groups in the study were not significantly different in terms of age, gender, smoking status, and serum level of folate and HbA1C (Table 1).

**Table 1:** Demographic data of patients.

Characteristics	Metformin group (n = 120)	Non-metformin group (n = 35)	*p*-Value
Age (years)	63.8 ± 6.9	65.9 ± 7.3	0.163
Male (female)	92 (28)	24 (11)	0.187
B12 (pg/ml)	268.5 ± 35.8	389.5 ± 29.8	0.029
Folate	5.3 ± 1.75	8. 2 ± 1.9	0.188
HbA1c (%)	7.4 ± 1.2	7.8 ± 1.8	0.215
MCV (μm^3^)	87.5 ± 2.9	83.7 ± 2.4	0.124
BMI (kg/m^2^)	31.17 ± 4.18	30.89 ± 6.18	0.185

Serum vitamin B12 levels ranged from 125 to 875 pg/ml. The mean serum vitamin B12 level ± SD was significantly lower in the metformin group (268.5 ± 35.8 vs. 389.5 ± 29.8 pg/ml, p = 0.029). Percentage of patients who had a definite and possible vitamin B12 deficiency was 45% and 29%, respectively. The patient group with metformin therapy had significantly higher prevalence of definite deficiency (32% vs. 9%, p < 0.02).

We found that the dose of metformin (1000 mg/day, orally) inversely affected serum vitamin B12 levels, within the group who were treated with metformin (n = 120), the mean serum vitamin B12 level was significantly lower in patients who were on metformin (175.2 ± 30.5 vs. 315.6 ± 37.8 pg/ml, p < 0.001).

Duration of metformin therapy was also found to affect the serum vitamin B12 levels. Patients on metformin therapy for less than five years (n = 73) had an average of serum vitamin B12 level of 276.9 ± 136.7 pg/ml, compared to an average of 222.4 ± 127.9 pg/ml (p < 0.001) in patients (n = 47) who had more than five years of treatment with metformin.

Serum folate concentration levels were found lower in metformin group but not significantly different (5.3 ± 1.75 vs. 8. 2 ±1.9 pg/ml, p = 0.213). MCV (μm^3^) levels were higher in metformin group (87.5 ± 2.9 vs. 83.7 ± 2.4).

## Discussion

Type 2 diabetes mellitus is a metabolic disorder that is characterized by high blood sugar (hyperglycemia) in the context of insulin resistance and relative lack of insulin [15, 16]. Whereas, in diabetes mellitus type 1 there is an absolute lack of insulin because of breakdown of islet cells in the pancreas [17, 18]. The symptoms are polydipsia, frequent urination, and constant hunger. Type 2 diabetes makes up about 90% cases of diabetes, with the other 10% due primarily to diabetes mellitus type 1 and gestational diabetes. Obesity is considered to be the primary cause of type 2 diabetes in people who are genetically predisposed to the disease [19].

Type 2 diabetes is initially treated by increasing exercise and lifestyle changes; if blood sugar levels do not decrease, then medications such as metformin or insulin are needed.

Metformin is an oral anti-diabetic drug of the biguanide class. American Diabetes Association suggests using metformin as the first-line medical therapy for type 2 diabetes mellitus [18]. It improves weight loss and the lipid profile and increases insulin sensitivity. Lactic acidosis in patient with renal failure, heart failure, and alcoholic patients are the most common side effects of metformin. Furthermore, it causes vitamin B12 deficiency. Treatment with metformin causing vitamin B12 malabsorption. Many studies have reported that 30% of patients have vitamin B12 malabsorption after treatment with metformin [20].

Biguanide interferes with an intrinsic factor-vitamin B12 complex and cubilin, an endocytic receptor involved in the absorption. The intrinsic factor-vitamin B12 complex is taken by the ileal cell surface, in a calcium dependent process, which may be affected by metformin through impaired calcium availability at the ileal level. Evidence to support this hypothesis is that dietary calcium supplementation reverses metformin-induced vitamin B12 malabsorption [21]. Biguanides such as metformin extend into the hydrocarbon core of the cell membrane through a hydrophobic tail. The protonated biguanide group gives a positive charge to the surface of the membrane, displacing divalent cations. Therefore, these kinds of drugs alter membrane potentials and affect their calcium-dependent function. Metformin has effect on the cubilin, which in turn, affects intrinsic factor-vitamin B12 complex absorption causing vitamin B12 deficiency.

In the present study, we found the prevalence of vitamin B12 deficiency among 28% patients with metformin therapy, this figure is higher than some of the previous studies have shown [7, 22].

The demographics and exclusion criteria used by previous studies are similar to those used in the current study but there is a difference in the method of data collection [7, 23].

In two separate studies by Ting et al. (2006) and Jolien et al. (2010), high metformin dosage (> 1.4 g/day) and long treatment duration (> 3 years) emerged as the most consistent risk factors of vitamin B12 deficiency for diabetic patients in the study population. It has also been shown that there is a significantly increased risk of vitamin B12 deficiency due to metformin treatment in diabetic patients with each additional 1 g/d dose increment of metformin [7, 8].

In this study, we found these parameters to be high risk factors for developing B12 deficiency [7]. Based on the data obtained from this study, a statistically significant inverse relationship between the duration of metformin use and serum vitamin B12 levels has been shown when comparing patients on metformin for five years or less to those taking it for more than five years. There was also a statistically significant association between vitamin B12 deficiency and dosage of metformin (lower the average serum vitamin B12 level, higher the dosage). This finding is similar to the findings by Ting et al. and Marar et al. [7, 24].

MCV is a well-known clinical parameter indicative of vitamin B12 deficiency. A case of megaloblastic anemia, secondary to vitamin B12 malabsorption and long-term metformin treatment has been reported [25]. In our study, there was no significant difference between the two groups in terms of hemoglobin, the MCV levels were higher in the metformin group but there was no significant difference.

The only source of vitamin B12 is food, therefore, diet has a key role in the prevention of its deficiency [18]. The method used in our study to estimate vitamin B12 intake is subject to error. Furthermore, some day-to-day variability in food intake is possible. Nutritional deficiency could have contributed to the low serum vitamin B12 levels in our patients.

## Limitations

We used serum vitamin B12 levels only to define deficiency. Metabolites (such as methylmalonic acid and total homocysteine) were not measured in our study. They are considered more sensitive indicators of vitamin B12 status than serum vitamin B12 levels. However, the assay for methylmalonic acid is costly, complex, and slow processing, and total homocysteine increases in patients with folate deficiency, which was taken into account for this study.

## Conclusion

At the end of this study, it is concluded that using metformin in type 2 diabetes mellitus treatment is significantly correlated with vitamin B12 deficiency. This supports other studies which state the long-term effect of metformin treatment for type 2 diabetics. This study also emphasizes baseline checking of vitamin B12 levels in patients on metformin therapy followed by serial monitoring of vitamin B12 levels as a routine in the diabetic patient’s healthcare system.

## Supporting information

S1 File Spreadsheet of the raw data. (XLSX)
